# Chasing Highs, Experiencing Lows: A Case of Hypokalemia Associated With Cannabis Use

**DOI:** 10.7759/cureus.83194

**Published:** 2025-04-29

**Authors:** Christopher T Gabbert, Fariha Bhuiyan, Terrance J Truitt

**Affiliations:** 1 Medical School, Edward Via College of Osteopathic Medicine, South Boston, USA; 2 Internal Medicine, Sentara Halifax Regional Hospital, South Boston, USA; 3 Pulmonary and Critical Care Medicine, Pulmonary Associates of Southside Virginia, South Boston, USA

**Keywords:** bradycardia, cannabis use, electrolyte disturbances, hypokalemia, intensive care unit stay

## Abstract

Hypokalemia is a relatively common reason for admission from the Emergency Department, with cannabinoid utilization being a more rare, indirect cause, as excessive use can lead to vomiting and diarrhea. More recently, there has been growing interest in the possible association between cannabis use and hypokalemia, even in the absence of gastrointestinal (GI) losses. This case involves a 24-year-old female with a history of anxiety, depression, and heavy cannabis use, who presented to the Emergency Department with bilateral lower extremity weakness and medial leg pain for two days. She was found to have an initial potassium level of 1.8, which prompted immediate repletion and admission to the ICU. Electrocardiogram (EKG) showed QT prolongation and bradycardia. Her hospital stay was complicated with a low phosphorus level of 1.0 after fluid administration. No additional renal abnormalities, nor identifiable causes for the hypokalemia, were identified following a comprehensive nephrological workup. The patient was discharged with a potassium level of 4.0 and instructions to follow up with nephrology and begin supplementation. This case underscores the importance of considering cannabinoid ingestion in the differential diagnosis of unexplained hypokalemia without GI manifestations.

## Introduction

Hypokalemia is a common electrolyte abnormality with a prevalence ranging from 2% to 11% in the general population, and even higher rates are observed in hospitalized patients [1,2]. Absolute potassium levels can decrease due to low dietary intake, renal losses, and gastrointestinal (GI) losses [1,2]. Additionally, serum potassium levels can fluctuate due to intercellular shifting, which occurs through several distinct mechanisms [2,3]. It is important to consider medications and substances that patients are taking, as hypokalemia can be a byproduct of side effects such as diuresis, vomiting, and diarrhea [2].

Cannabinoid products are now recognized as contributors to GI-loss-related hypokalemia due to the growing prevalence and recognition of cannabinoid hyperemesis syndrome (CHS) [4]. However, cannabinoid-induced hypokalemia remains a rare cause for hypokalemia overall, particularly when GI loss is not a participating factor [4]. This case will discuss a relatively healthy 24-year-old female who was hospitalized with severe hypokalemia, independent of GI loss, following chronic, heavy cannabis use.

## Case presentation

A 24-year-old female with a past medical history of depression, anxiety, and cannabis use presented to the emergency department with a complaint of lower extremity weakness of two days duration. She first noticed the weakness following a mechanical fall, with no loss of consciousness, no syncope, and no injury. The weakness progressed to a point where she could no longer ambulate. She reported palpitations and myalgias (particularly in the medial aspects of both legs), but denied fever, chills, nausea, vomiting, dizziness, lightheadedness, headaches, recent changes to activity, changes to medications or diet, intake of high carbohydrate meals, and any previous instances of weakness.

The patient’s active medications included 100 mg of oral bupropion daily, 20 mg of oral escitalopram daily, 20 mg of oral propranolol as needed, and an oral, daily two-hormone contraceptive. Although the patient had no remarkable family history or allergies, her social history was significant for intermittent tobacco use, alongside daily cannabis use via vaporizer pen since age 18. Of note, the patient reported finishing one vaporizer cartridge per day; when asked about product usage guidelines, the patient disclosed that each cartridge should last approximately three months with expected, moderate use.

Initial physical exam revealed normal heart rate and heart sounds; cranial nerves were intact; bilateral 1/5 strength for dorsiflexion of ankles, 5/5 strength for ankle plantarflexion, hip flexion, and knee flexion; intact sensation to the lower extremities; and palpable distal pulses. Ankle deep tendon reflexes (DTR) were not tested, as the patient’s feet were significantly plantarflexed at rest. Passive movement of the ankles demonstrated no resistance. DTR of bilateral knees were +1. Initial vital signs included a heart rate of 60 beats per minute, blood pressure of 121/86, respiratory rate of 14, oxygen saturation of 94%, and oral temperature of 98.7℉. The patient’s body mass index (BMI) was 36.12.

**Figure 1:**
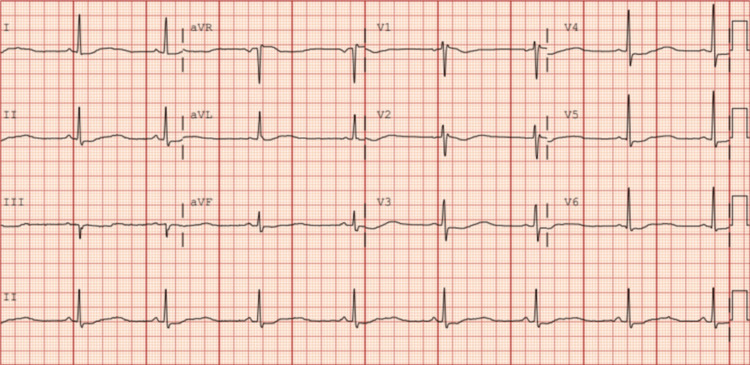
Electrocardiogram on admission Diffuse QT prolongation most apparent in lead II.

Initial laboratory results were remarkable for a potassium value of 1.8, with stable magnesium and calcium (Table 1). An electrocardiogram (EKG) was obtained, revealing sinus bradycardia with a prolonged corrected QT (QTc) interval of 628 milliseconds, as measured by Fridericia’s formula (Figure 1). The patient was given 40 milliequivalents (mEq) of oral potassium and 10 mEq of intravenous (IV) potassium. One gram of oral magnesium was also provided for cardiac membrane stabilization. The patient’s escitalopram and propranolol were placed on hold, given her prolonged QTc interval and bradycardia, respectively. She was then promptly transferred to the intensive care unit for close cardiac and electrolyte monitoring.

**Table 1:** Initial laboratory results Phosphorus gathered following aggressive fluid administration.

Test	Result	Normal Range
White Blood Cell (WBC)	13.9	4.0 – 11.0 K/uL
Hemoglobin (Hgb)	13.1	11.7 – 15.5 g/dL
Platelet (Plt)	414	140 – 440 K/uL
Sodium (Na)	141	133 – 145 mmol/L
Potassium (K)	1.8	3.5 – 5.5 mmol/L
Chloride (Cl)	107	98 – 110 mmol/L
Bicarbonate (CO_2_)	19	20 – 32 mmol/L
Anion Gap	15	3.0 – 15.0 mmol/L
Albumin	4.4	3.5 – 5.0 g/dL
Blood Urea Nitrogen (BUN)	8	6 – 22 mg/dL
Creatinine (Cr)	0.6	0.5 – 1.2 mg/dL
Random Blood Glucose	102	70 – 99 mg/dL
Calcium (Ca)	10.2	8.4 – 10.5 mg/dL
Magnesium (Mg)	1.8	1.6 – 2.5 mg/dL
Phosphorus (PO_4_)*	1.0	2.4 – 4.7 mg/dL
Urine Cannabinoid Screen	Detected	Detection Threshold 50 ng/mL

Following transfer and extensive fluid administration, the patient was also found to have a phosphorus value of 1.0, prompting IV repletion (Table 1). The remainder of the patient’s hospital stay was unremarkable. On day 2 of admission, renal studies found no increase in urine potassium or serum pH imbalance; thyroid panel was unremarkable; and electrolytes were stable throughout admission following hypokalemia correction (Table 2). The patient was transferred to a general medical floor with telemetry on day 3 of admission, where cardiac monitoring demonstrated normal sinus rhythm; she also had complete alleviation of weakness on the same day. On day 4, she was discharged with a final potassium value of 4.0 mmol/L and instructed to supplement with a 10 mEq oral potassium daily; she also had a follow-up appointment one week later at a nephrology clinic, with repeat laboratory work at which time her potassium value was 4.0 mmol/L.

**Table 2:** Laboratory values including renal function tests Some values have no normal range given collection at a nonstandard time. These values were interpreted within a clinical context.

Test	Result	Normal Range/Units
Serum Osmolality	293	280-300 mOs/kg
Urine Creatinine	28	mg/dL
Urine Sodium	52	mmol/dL
Urine Potassium	<7	mmol/dL
Urine Chloride	56	mmol/dL
Urine Osmolality	142	200 – 1200 mOs/kg
Urine pH	7.5	5.0-8.0 pH
Bicarbonate (CO_2_)	23	20-32 mmol/L
Anion Gap	12	3.0-15.0 mmol/L
Thyroid Stimulating Hormone (TSH)	1.84	0.27-4.20 mcU/mL
Thyroxine (T4)	7.6	4.5-10.9 mcg/dL
Triiodothyronine (T3)	126	80-200 ng/dL
Aldosterone	<1	ng/dL
Cortisol	20.0	mcg/dL

## Discussion

With the use of cannabinoid-containing products becoming increasingly commonplace, it is important to thoroughly investigate their acute/chronic effects in order to mitigate unintended consequences and educate users overall. There are few literature reports of severe hypokalemia associated with natural and synthetic cannabinoid use; reasons for such an association are still relatively unclear, especially in the absence of GI losses [5-8].

In this case, the patient denied vomiting and diarrhea, which are both commonly associated with heavy cannabis use and CHS [4]. Additionally, although the literature suggests that increased appetite after cannabinoid ingestion may trigger a spike in insulin secretion, leading to acute intracellular potassium shifting, her clinical history did not support this [5]. Her abrupt onset of musculoskeletal symptoms aligns with hypokalemic periodic paralysis (HPP), with cannabis being a possible instigating factor [5,9]. The patient’s age fell within the expected range for initial onset, and her condition improved with potassium replacement, as seen in HPP [9]. HPP is typically inherited in an autosomal dominant pattern and presents with proximal muscle weakness; however, the patient’s negative family history and the lack of proximal muscle weakness reduce the likelihood of this diagnosis. Furthermore, given the absence of typical triggers like increased carbohydrate consumption or recent physical exertion, the most probable explanation for her hypokalemia is cannabis use, as opposed to an exacerbation of HPP [9]. 

Endocannabinoid receptors are commonly expressed in renal tissues, suggesting that exogenous cannabinoids can exert a direct, and possibly detrimental, effect on kidney function [10]. This is further supported by a systematic review that demonstrates an association between cannabinoid use and acute kidney injury (AKI) [11]. However, the patient in this case did not meet criteria for AKI and showed no signs of renal toxicity during hospitalization. Notably, one study reported a statistically significant difference in potassium levels between chronic cannabis users and non-users, which may account for both this patient’s condition and a possible chronic effect of cannabis use on kidney function [12].

Ultimately, this patient’s hypokalemia could not be attributed to other common causes. Her cortisol and aldosterone levels were normal, and none of her medications were known to cause hypokalemia. Her urinary potassium demonstrated a proper compensatory decrease while her creatinine was normal, suggesting that renal loss of potassium was improbable. GI loss was also improbable, given the absence of reported vomiting and diarrhea along with relatively normal chloride and bicarbonate values. Additionally, her lack of chronic alcohol use and anorexia made malnutrition and low dietary intake unlikely.

For more rare causes, renal tubular acidosis type I is one to consider, given her normal urinary pH, low potassium, and mild decrease in bicarbonate on presentation [13]. However, her correction of bicarbonate with only normal saline, along with her lack of history of childhood symptoms such as failure to thrive, rickets, and kidney stones, makes this diagnosis unlikely [13]. Bartter and Gitelman syndromes can also be considered, although her normal aldosterone and adult-onset presentation of these electrolyte abnormalities make these diagnoses unlikely [14]. Lastly, her thyroid-stimulating hormone (TSH) was normal, ruling out thyrotoxic periodic paralysis [9].

## Conclusions

While the management of laboratory-confirmed hypokalemia is standard, regardless of cause, identifying the underlying etiology can help avoid unnecessary healthcare costs and ensure appropriate treatment to prevent recurrence. This case emphasizes the need for further research into the relationship between cannabinoids and electrolyte disturbances, beyond the often-considered mechanisms of transient hyperinsulinemia and intractable vomiting/diarrhea. The exact mechanism behind this association remains unclear, prompting further investigation. Raising awareness about this effect may help prevent life-threatening consequences of hypokalemia.
